# Pediatric formulations in national essential medicines lists: a cross-sectional study

**DOI:** 10.3389/fped.2025.1566841

**Published:** 2025-05-02

**Authors:** Camila Heredia, Moizza Zia Ul Haq, Bernadette Cappello, Farihah Malik, Martina Penazzato, Lorenzo Moja, Navindra Persaud

**Affiliations:** ^1^ MAP Centre for Urban Health Solutions, Li Ka Shing Institute, St. Michael’s Hospital, Unity Health Toronto, Toronto, ON, Canada; ^2^ Department of Health Products Policy and Standards, World Health Organization, Geneva, Switzerland; ^3^ University Institute of Public Health, The University of Lahore, Lahore, Pakistan; ^4^ Science Division, Research for Health Department, World Health Organization, Geneva, Switzerland; ^5^ Department of Family and Community Medicine, Faculty of Medicine, University of Toronto, Toronto, ON, Canada

**Keywords:** pediatric formulations, essential medicines, essential medicine lists, pediatric lists, children

## Abstract

**Background:**

Children need specialized medicine formulations for proper dosing, safety, and adherence. The World Health Organization developed a pediatric essential medicines list prioritising pediatric formulations. Given global commitment by countries to children's health, we expected national lists similarly prioritizing pediatric medicines and formulations. We assessed the extent to which national lists include medicines for children.

**Methods:**

We used a global essential medicines database to collect data on national lists identifying pediatric lists, medicines, and formulations. Six key therapeutic areas were selected *a priori* to present granular data. Data were categorized by country characteristics and income levels.

**Results:**

Our study found that most countries do not include pediatric formulations in their Essential Medicine Lists (EMLs), especially high-income European countries. Of the 22 countries that do, most list medicines for infections, antiretrovirals, and cancer, but gaps exist for antitrypanosomal, antileishmanial, and antihepatitis treatments. Paracetamol had the most diverse formulations. Additionally, differences were found between national and World Health Organization (WHO) EMLs, with some countries listing fewer medicines overall, though some countries included more treatments for HIV and hepatitis than the WHO Essential Medicines List for children (EMLc).

**Conclusion:**

In many countries, it is unclear which medicines for children are prioritized, if any. The problem is particularly acute in high-income countries. Misalignments between national lists and the World Health Organization are common. There is little evidence that countries are adequately implementing medicines policies for youth.

## 1 Introduction

Children often have therapeutic needs that require dedicated medicines and formulations to maximize effectiveness, appropriate dosing limiting toxicity, and to promote adherence (1–3). Ethical and logistic concerns, and commercial causes such as a relatively small “market” for pediatric medicines, have been proposed as reasons behind scarce attention to children needs (1). Developing pediatric formulations often requires additional research, clinical trials, and smaller production runs, making the process expensive. Additionally, strict regulatory requirements for safety and efficacy in children can make bringing these medicines to market more complicated and costly, resulting in a smaller market that may not attract the same level of commercial investment as adult medicines (1). To increase attention to needs of young patients, the World Health Organization (WHO) developed a dedicated version of the model list of Essential Medicines (EML) for children (WHO EMLc). The EMLc follows the same aim and process as the master EML: launched in 2007, it is updated and published every two years, intended as a guide for countries or regional authorities to prioritize access to safe, effective, high-quality and cost-effective essential medicines and vaccines to address public health priorities (4). Essential medicines are a central component of Universal Health Coverage, indicating where health funding should be primarily directed (5).

Although high-level statements about prioritizing children's health and therapies are very common these days (6), little is known about the extent to which national EMLs include a companion list dedicated to children or pediatric formulations, a first step to ensure availability of therapeutics dedicated to children. Global initiatives emphasize children's health as a fundamental right, with the UN Convention on the Rights of the Child (CRC) mandating access to healthcare, nutrition, and a safe environment. Aligned with this, the WHO Global Strategy, Sustainable Development Goals (SDGs), and the Global Action Plan for Healthy Lives and Well-being for All call for reducing child mortality, improving well-being, and ensuring equitable healthcare through coordinated global efforts (6–9). Some data on pediatric medicines and formulations come from studies conducted in single countries - Serbia, Mongolia and Thailand - with far from encouraging results. Research from Serbia highlights a critical shortage of pediatric-suitable formulations, emphasizing the global disparities in children's access to essential medicines. Studies in Mongolia and Thailand further reveal challenges in procurement, distribution, and effective use, underscoring the need for national policies, such as a dedicated Pediatric Medicine List, to improve availability, quality, and acceptability of medicines for children (10–12). We systematically analyzed handling of medicines for children and pediatric formulations within national EMLs, providing a snapshot of the role given to these items in an important medicine policy tool adopted by most countries globally.

## 2 Materials and methods

We employed the Global Essential Medicines database of national EMLs that was updated in November of 2023 to collect information on companion lists explicitly dedicated to children and pediatric formulations (13). Briefly, the database was created by searching for national EMLs through various approaches: searches on government (e.g., Ministry of Health) and medicine regulatory agency websites, searches in search engines (e.g., Google), and contacting WHO technical regional and country officers responsible for access to medicine policies. We also compared retrieved national EMLs with those referenced by studies using national EMLs, identified in Medline or through the WHO network of experts conducting research on access to medicines. Two researchers abstracted data from each national EML. Disagreements were solved by consensus or by involving a third researcher. An algorithm was used to translate some medicine names and to assign ATC (Anatomical Therapeutic Chemical) codes. The database consists of a matrix listing each medicine and each country and indicates which countries list which medicines. We treated formulations with the same concentration as the same even if they were expressed in different units (e.g., 1 mg/ml and 5 mg/5 ml were considered the same). We finally contrasted all items recommended by the 2023 version of the WHO EMLc to items recommended in national EMLs, measuring the percentage of agreement.

Country characteristics were analyzed to assess their influence on the creation of essential medicine lists by determining healthcare demand, disease burden, financial commitment to healthcare, and capacity for procuring and distributing essential medicines. Population size, was obtained from the United Nations Population Data (14), life expectancy was obtained from the Central Intelligence Agency (15), and health expenditure data was obtained from the Global Health Observatory (16), except for Somalia and the Democratic People's Republic of Korea (17, 18). Countries' income levels were extracted from World Bank (19). Most of the data pertained to the year 2023; if 2023 records were unavailable, information from the nearest available year to 2023 was accessed.

Data were tabulated according to country WHO regions (20) and World Bank income classification. Lists that included pediatric formulations for less than ten medicines, were included in the descriptive analysis but excluded from inferential analysis because inclusion of children medicines might have been accidental rather than planned.

Before starting the study, we selected six therapeutic areas of interest, in line with the priority areas identified by the Global Accelerator for Paediatric Formulations Network (GAP-f) (21): antibiotics, cancer treatment, tropical neglected diseases, human immunodeficiency virus (HIV), tuberculosis, and viral hepatitis. Medicines for these areas were identified based on the ATC code; antibiotics were categorised following the WHO AWaRe classification (22, 23). Subgroup analyses referring to these areas are presented in the Supplementary Appendix section.

## 3 Results

**Figure 1:**
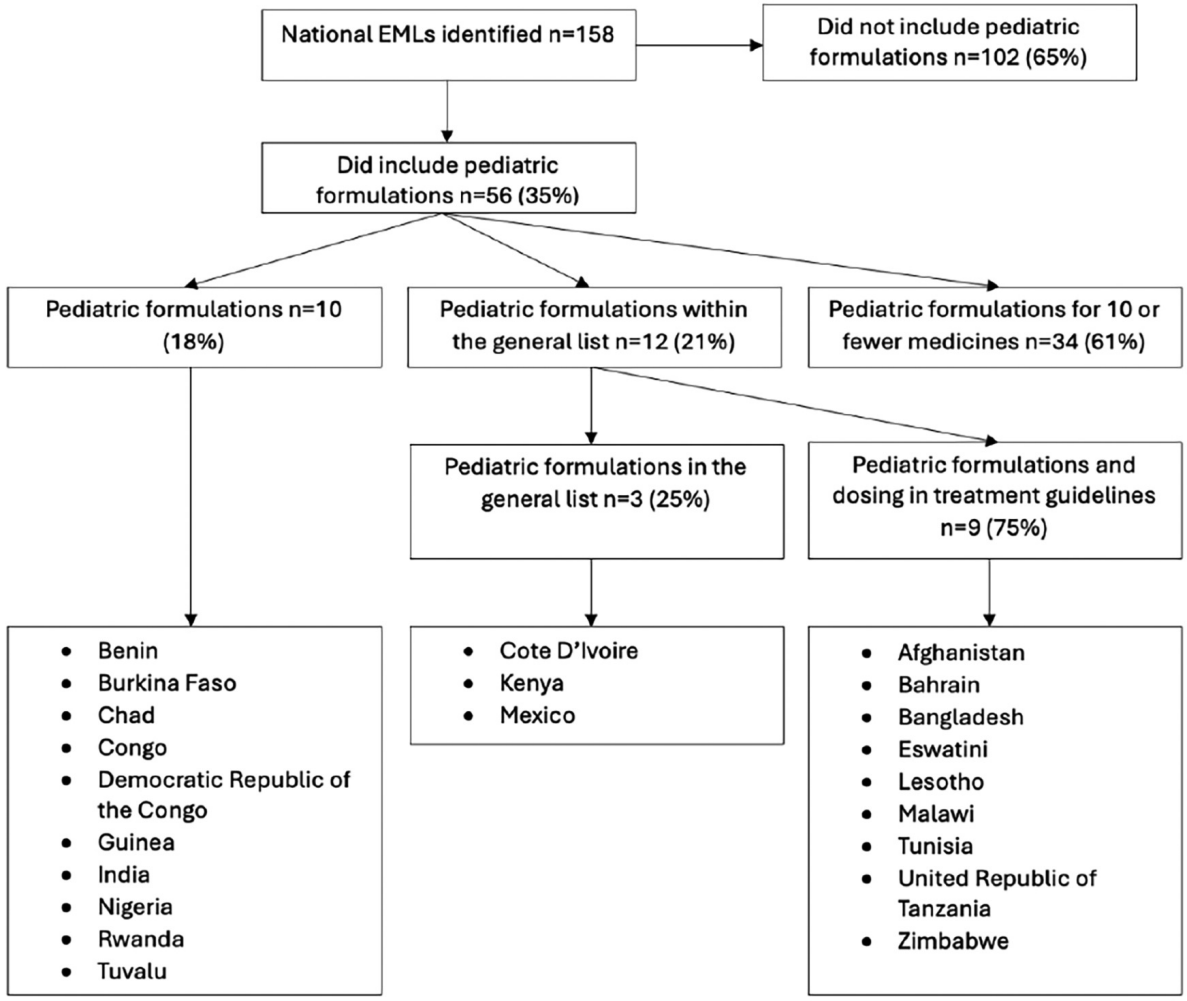
Inclusion of paediatric formulations in EMLs.

National EMLs were available for 158 countries. Ten (6.3%) had separate lists for children that specified pediatric formulations. Another 46 lists (29.1%) included pediatric formulations: 34 (73.9%) included pediatric formulations for a small number (ten or fewer) of medicines and 12 (26.1%) included pediatric formulations within the general list (Figure 1). In total, 22 (13.9%) countries prioritized medicines and formulations for children. Medicines and formulations for children were most often included in national EMLs from countries in the African region (*n* = 15 out of 47 countries, 31.9%), followed by the Eastern Mediterranean region (*n* = 3 out of 21 countries, 14.3%) (Table 1). No European country had a separate pediatric EML or included pediatric formulations on their EMLs. Pediatric medicines and formulations were most commonly included by countries categorised as lower middle-income level (*n* = 13 out of 54 countries; 24.1%) followed by low-income level (*n* = 6 out of 26 countries, 23.1%) (Table 1). Overall, these 22 countries presented considerable disparity in population size, life expectancy, and health expenditure, with the majority having a per capita health expenditure lower than 200 US$ (Table 2) (15).

**Table 1:** Summary of national EMLs reviewed, by WHO region and world bank income classification (extracted from website on July 2024) (19, 20).

Country classifications	All member states	Countries for which national EML available	Pediatric formulations in separate list	Pediatric formulations within general list	Pediatric formulations for ≤10 medicines
	A	B (as % of A)	C (as % of B)	D (as % of B)	E (as % of B)
Total	194	158 (81%)	10 (6%)	12 (8%)	34 (22%)
By WHO region:					
Africa	47	47 (100%)	8 (17%)	7 (15%)	8 (17%)
Americas	35	30 (86%)	0	1 (3%)	5 (17%)
Eastern Mediterranean	21	18 (86%)	0	3 (17%)	6 (33%)
Europe	53	32 (60%)	0	0	4 (13%)
South-East Asia	11	11 (100%)	1 (9%)	1 (9%)	4 (36%)
Western Pacific	27	20 (74%)	1 (5%)	0	7 (35%)
By World Bank income level:					
High income	59	34 (58%)	0	1 (3%)	6 (18%)
Upper middle income	52	50 (96%)	1 (2%)	1 (2%)	13 (26%)
Lower middle income	54	46 (85%)	5 (11%)	8 (17%)	8 (17%)
Low income	26	26 (100%)	4 (15%)	2 (8%)	6 (23%)
Unclassified	3	2 (67%)	0	0	1 (50%)

**Table 2:** List of the 22 countries listing pediatric formulations and their characteristics.

Country	Separate pediatric list	Year of EML – Year of EMLc	Total n° of medicines in general list	Total n° pediatric medicines	Total n° pediatric formulations	Population size 2021 est. (14)	Life expectancy 2023 est. (15)	Health expenditure (US$ per capita) 2021 (15)	World Bank income level (19)
WHO model EML and EMLc	Yes	2023–2023	534	368	792	–	–	–	–
Afghanistan (AFG)	No	2015–2015	296	175	400	40,099,462	54.1	81	Low
Bahrain (BHR)	No	2015–2015	562	200	324	1,463,266	80.1	1,146	High
Bangladesh (BGD)	No	2019–2019	254	118	265	169,356,251	75	58	Lower middle
Benin (BEN) *	Yes	2018–2018	396	357	719	12,996,895	62.6	35	Lower middle
Burkina Faso (BFA) *	Yes	2020–2020	379	258	509	22,100,684	63.8	57	Low
Chad (TCD) *	Yes	2022–2022	431	280	547	17,179,740	59.6	36	Low
Congo (COG) *	Yes	2019–2019	339	198	383	5,835,806	72.2	81	Lower middle
Cote D’Ivoire (CIV)	No	2020–2020	577	64	85	27,478,249	62.7	82	Lower middle
Democratic Republic of the Congo (COD) *	Yes	2020–2020	355	251	544	95,894,119	62.2	22	Low
Eswatini (SWZ)	No	2012–2012	312	83	135	1,192,271	60.2	280	Lower middle
Guinea (GIN) *	Yes	2021–2021	305	232	380	13,531,906	64.3	45	Lower middle
India (IND) *	Yes	2022–2011	350	119	265	1,407,563,842	67.7	74	Lower middle
Kenya (KEN)	No	2019–2019	485	65	74	53,005,614	70	95	Lower middle
Lesotho (LSO)	No	2005–2005	190	67	91	2,281,455	59.9	115	Lower middle
Malawi (MWI)	No	2015–2015	333	124	177	19,889,742	72.7	47	Low
Mexico (MEX)	No	2017–2017	794	369	538	126,705,138	73.5	611	Upper middle
Nigeria (NGA) *	Yes	2020–2020	405	283	617	213,401,323	61.8	84	Lower middle
Rwanda (RWA) *	Yes	2022–2022	393	271	442	13,461,888	66.2	60	Low
Tunisia (TUN)	No	2012–2012	642	46	54	12,262,946	77.1	265	Lower middle
Tuvalu (TUV) *	Yes	2010–2010	179	126	171	11,204	68.7	1071	Upper middle
United Republic of Tanzania (TZA)	No	2021–2021	452	166	238	63,588,334	70.5	37	Lower middle
Zimbabwe (ZWE)	No	2020–2020	301	119	185	15,993,524	66.8	63	Lower middle

For some countries, the medicines listed for children are also listed for adults (and thus, the numbers in the two lists for those countries are similar). Combination of two or more medicines in the adult EML were divided, whereas pediatric medicines were treated as unified combinations, potentially leading to a slight variance in overall numbers. Countries with an asterisk are the ones with separate pediatric lists.

### 3.1 Pediatric medicines and formulations listed

**Figure 2:**
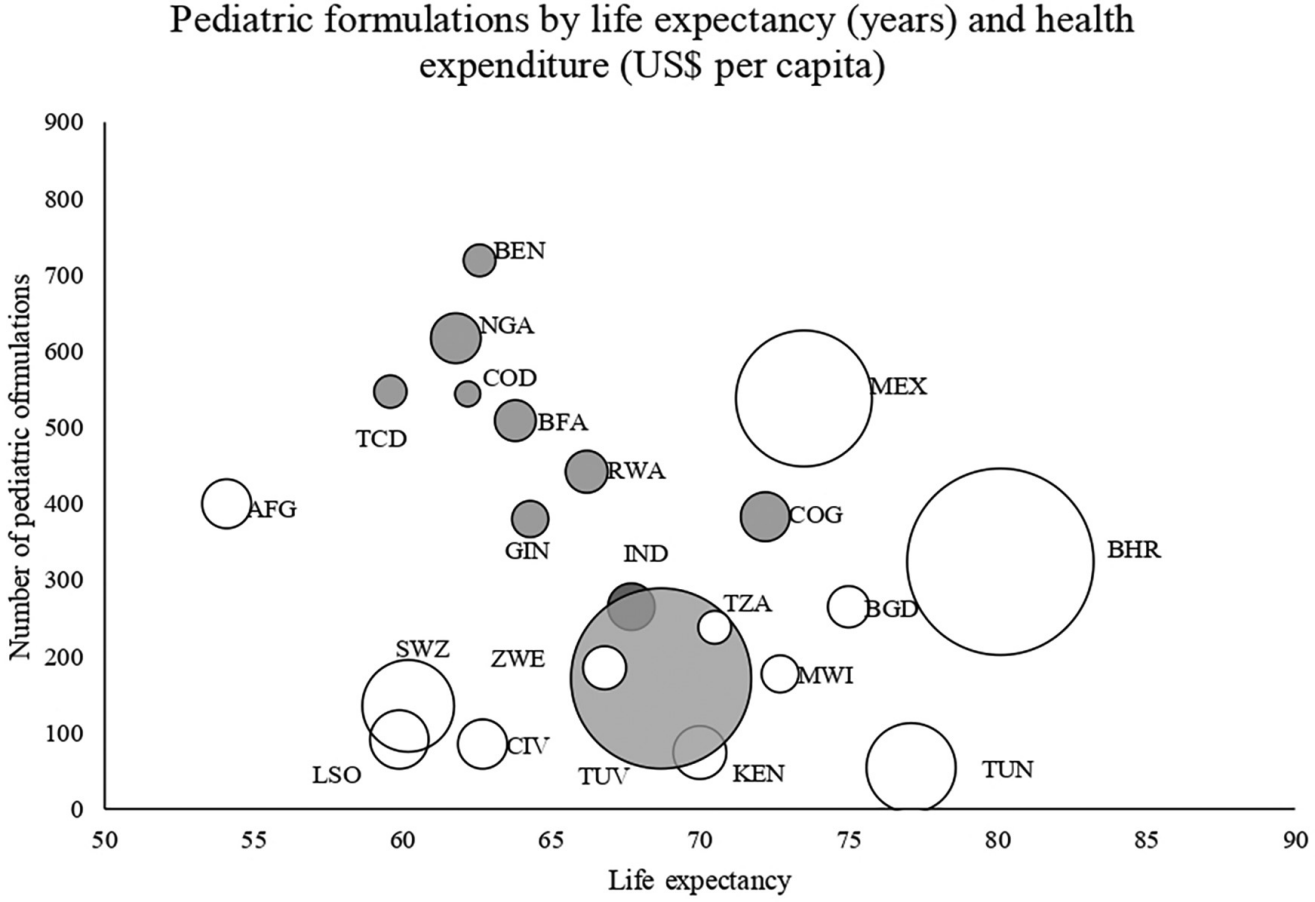
Number of pediatric formulation listed by life expectancy (years) and health care expenditure per capita (represented by bubble size) (12). In grey, countries with specific pediatric lists.

From the 22 countries listing pediatric formulations and medicines in either separate lists (*n* = 10) or general lists (*n* = 12), the number of pediatric medicines ranged from 46 (Tunisia) to 369 (Mexico), with a median of 170 (IQR 118–256), compared to the 2023 WHO EMLc listing 368. The number of pediatric formulations ranged from 54 (Tunisia) to 719 (Benin), median 294 (IQR 172–492), while the WHO EMLc included 792. The 22 analysed lists were published over a long period, spanning from 2005 (Lesotho) to 2022 (Chad and Rwanda). From the ten countries with a specific EML for children, nine published their national EML and national EMLc in the same year. India had a gap of 11 years between the latest EML update (2022) and the latest EMLc (2011) (Table 2). Countries that include pediatric formulations into their general lists (white bubbles, Figure 2) tended to have a lower number of pediatric formulations, to have higher per capita health spending, and longer life expectancies compared to those with separate pediatric lists (grey bubbles, Figure 2).

The number of medicines included in general national EMLs was not always related with the number of pediatric medicines, as some countries like Kenya and Tunisia showed disparities in the number of medicines for adults compared to the number of medicines for children in their pediatric section (e.g., Kenya with 485 medicines for adults and only 65 for children, or Tunisia listing 642 and 46 respectively). Other countries like Benin and Tuvalu included a similar number of medicines for adults and for children (e.g., Benin listing 396 and 357 respectively, or Tuvalu with 179 medicines for adults and 126 for children) (Table 2).

From all the pediatric formulations listed in the 22 EMLs, paracetamol (acetaminophen) was the individual medicine with the higher diversity of formulations listed (28 different formulations, e.g., injection, oral liquid, powder, rectal dosage, and solid oral dosage forms such as tablet and capsule). Benin's list featured the highest number of distinct paracetamol formulations, totalling ten.

### 3.2 Comparison with the WHO EMLc

**Figure 3:**
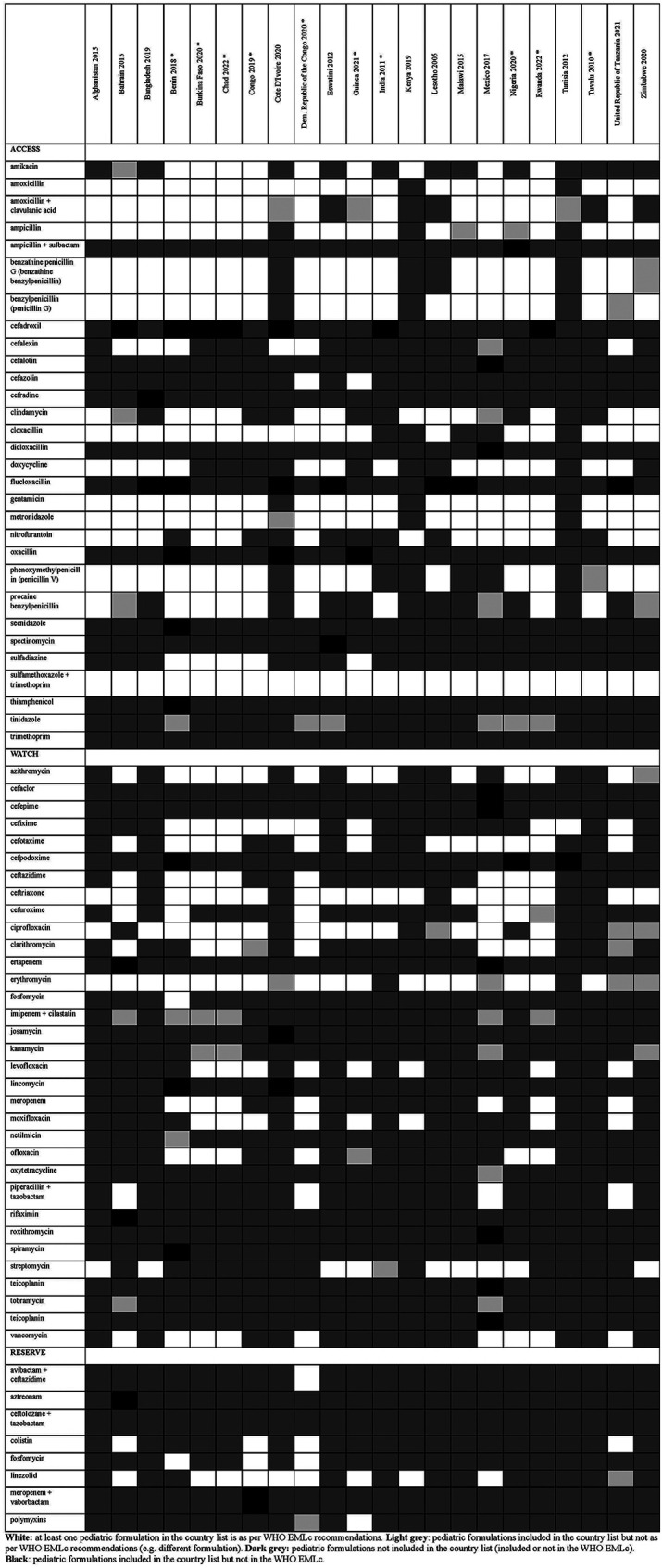
Status of inclusion of antibiotic formulations in the 22 lists. Countries with an asterisk are the ones with separate pediatric lists.

**Figure 4:**
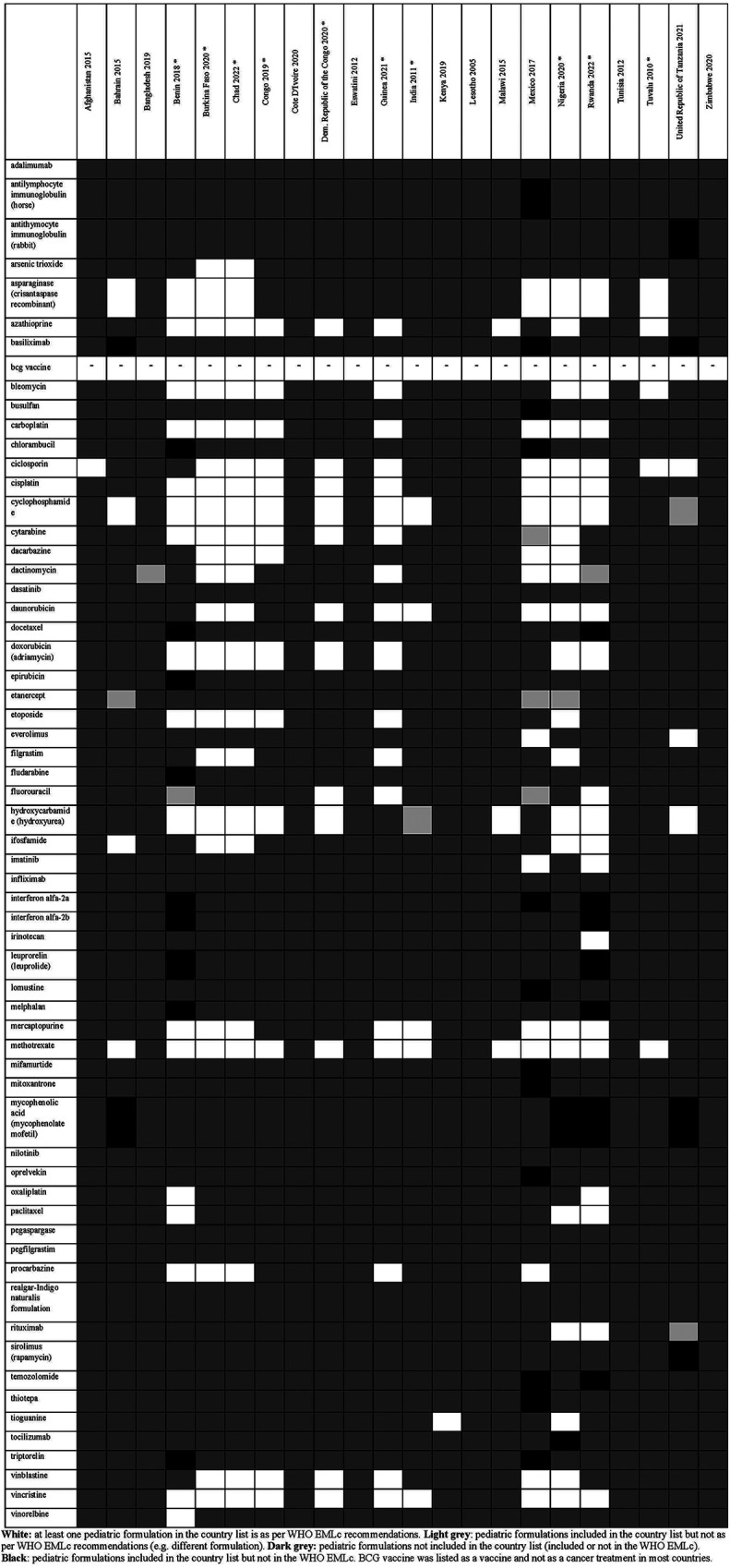
Status of inclusion of cancer formulations in the 22 lists. Countries with an asterisk are the ones with separate pediatric lists. BCG vaccine was listed as a vaccine and not as a cancer treatment in most countries.

**Figure 5:**
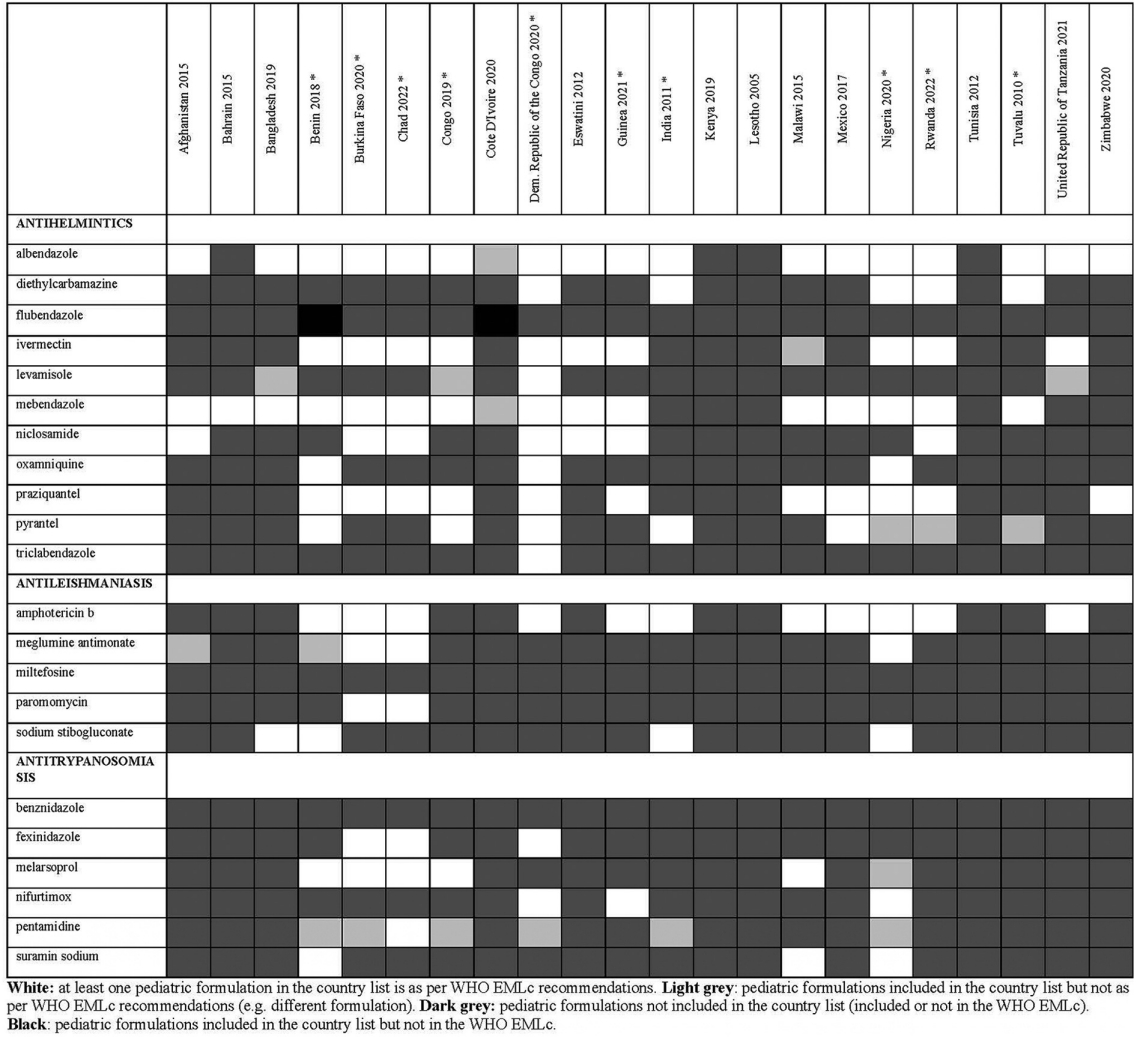
Status of inclusion of antihelmintics, antileishmaniasis, and antitrypanosomal formulations in the 22 lists. Countries with an asterisk are the ones with separate pediatric lists.

**Figure 6:**
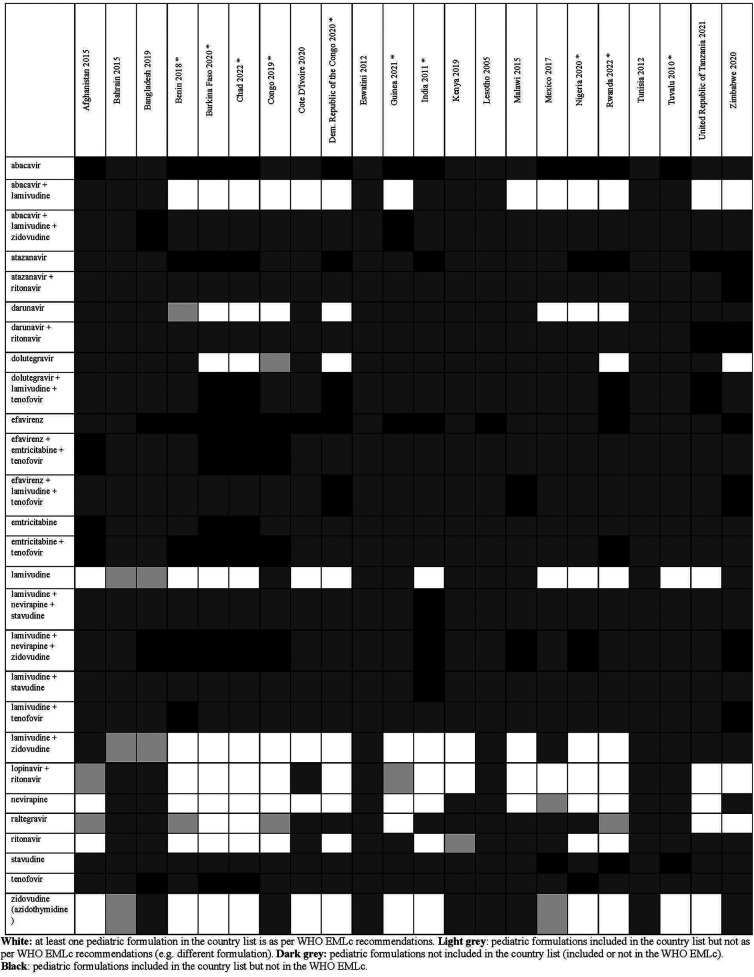
Status of inclusion of HIV formulations in the 22 lists. Countries with an asterisk are the ones with separate pediatric lists.

**Figure 7:**
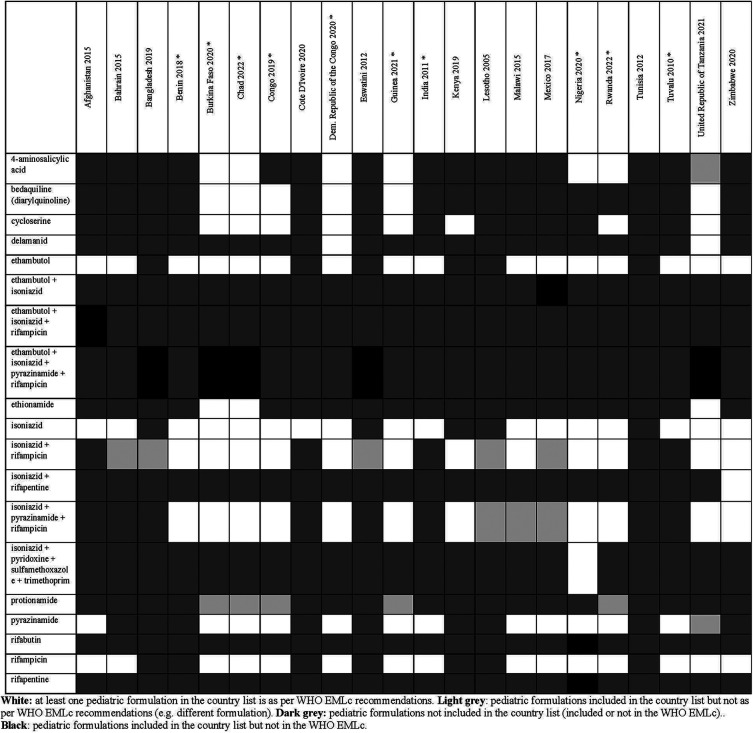
Status of inclusion of tuberculostatic formulations in the 22 lists. Countries with an asterisk are the ones with separate pediatric lists.

**Figure 8:**
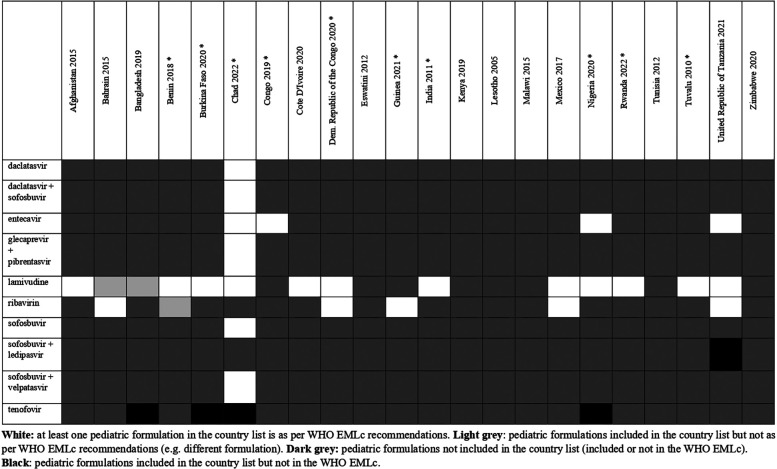
Status of inclusion of hepatitis formulations in the 22 lists. Countries with an asterisk are the ones with separate pediatric lists.

Overall, there were 953 formulations (comprising 860 individual formulations and 93 combinations) listed by at least one country and not included in the WHO EMLc. Some countries listed formulations of medicines for which the WHO EMLc does not provide specific formulation information (since the WHO EMLc mentioned these medicines as therapeutic alternatives to other medicines using the “square box”, Table 3). Examples include 16 countries listing activated charcoal powder formulations (Afghanistan, Benin, Burkina Faso, Chad, Congo, Democratic Republic of the Congo, Eswatini, Guinea, India, Malawi, Mexico, Nigeria, Rwanda, Tuvalu, United Republic of Tanzania and Zimbabwe), and five countries listing mometasone nasal spray formulation (Benin, Congo, Guinea, Mexico, and United Republic of Tanzania). For the 22 countries that specify pediatric formulations, the analyses of the six therapeutic subgroups (see Figures 3–8 and Supplementary Appendix), revealed significant discrepancies between national essential medicine lists and the WHO Essential Medicines List for Children (WHO EMLc). ACCESS group antibiotics were the most frequently listed medicines, with Benin including the highest number, while cancer treatments showed the greatest gaps between country lists and WHO recommendations. Some countries, such as Chad and Burkina Faso, listed more HIV and tuberculosis treatments than the WHO EMLc, while others, like Tunisia, had minimal representation across all areas. Additionally, 69 medicines were cited by at least one country but not included in the WHO EMLc, with efavirenz being the most commonly mentioned despite its removal from the WHO list in 2021. The findings highlight inconsistencies in pediatric medicine availability and the need for greater alignment with global recommendations to ensure comprehensive treatment access for children.

**Table 3:** Formulations included in the 22 national lists for medicines listed by the WHO EMLc as therapeutic alternatives without specified formulations.

Medicine listed in WHO EMLc with no specified formulation	Formulations listed by countries [number of countries listing them]
Activated charcoal (powder)	Powder 100 mg [6], 25 g [4], 50 g [3], 150 mg [1], 200 mg [1], and 5 g [1].
Amikacin (ophthalmological preparation)	No country listing ophthalmological preparations.
Anidulafungin	No country listing the medicine.
Atracurium	Injection 10 mg/ml (includes 250 mg/2.5 ml and 250 mg/25 ml) [4].
Beclometasone	Inhalation 50 ug/dose [11], and 250ug/dose [4].
Calcitriol	Solid oral dosage form 0.25 ug [2]. No country listing cream, ointment, or lotion.
Carbimazole	Solid oral dosage form 5 mg [8], and 20 mg [3].
Caspofungin	No country listing the medicine.
Cetirizine	Solid oral dosage form 10 mg [4], and oral liquid 5 mg/5 ml [1].
Chlorothiazide	No country listing the medicine.
Chlortalidone	Solid oral dosage form 50 mg [1].
Chlortetracycline	No country listing the medicine.
Ciclesonide	No country listing the medicine.
Dalteparin	No country listing the medicine.
Darbepoetin alfa	Injection 10 ug/0.4 ml [2], 20 ug/0.5 ml [2], 60 ug/o.3 ml [2], 30 ug/0.3 ml [1], 40 ug/0.4 ml [1], 100 ug/0.5 ml [1], and 300 ug/0.6 ml [1].
Deferiprone	No country listing the medicine.
Dinoprostone (prostaglandin e2)	Injection 1 mg/ml [4].
Dolasetron	No country listing the medicine.
Erythromycin	Oral liquid 125 mg/5 ml [14], solid oral dosage form 250 mg [13], oral liquid 250 mg/5 ml [7], solid oral dosage form 500 mg [7], eye ointment 0.5% [6], oral liquid 200 mg/5 ml [2], injection 1 g [1], oral liquid (drops) 50 mg/1.25 ml [1], oral liquid 100 mg/5 ml [1], solid oral dosage form 200 mg [1], and 400 mg [1].
Etanercept	Injection 25 mg/0.5 ml [3].
Fexofenadine	Solid oral dosage form 120 mg [1], and 180 mg [1].
Flunisolide	No country listing the medicine.
Fluticasone	No country listing the medicine.
Granisetron	Injection 3 mg [2], and 1 mg [1], and oral liquid 20 mg/100 ml [1].
Hydromorphone	No country listing the medicine.
Imipenem + cilastatin	Injection 500 mg [5], and 250 mg [3].
Indometacin	Eye drops 0.1% [3]. No country listing injection.
Infliximab	No country listing the medicine.
Insulin degludec	One country listing, no formulation.
Insulin detemir	Injection 100 IU/ml [1].
Insulin glargine	Injection 100 IU/ml [1], and 300 IU [1].
Kanamycin	Injection 1 g [4]. No country listing eye drops.
Methoxy polyethylene glycol-epoetin beta	No country listing the medicine.
Mometasone	Nasal spray 50 ug [5].
Multienzymes (pancreatic enzymes)	Solid oral dosage form 150 mg to 300 mg [2].
Nadroparin	No country listing the medicine.
Netilmicin	Injection 25 mg [1], and 50 mg [1]. No country listing eye drops.
Oxycodone	No country listing the medicine.
Oxytetracycline	Injection 100 mg [1]. No country listing eye ointment.
Palonosetron	No country listing the medicine.
Prednisone	Solid oral dosage from 20 mg [5], 5 mg [2], 1 mg [1], and 50 mg [1].
Protionamide	Solid oral dosage form 250 mg [4].
Tacalcitol	No country listing the medicine.
Terbutaline	Injection 0.5 mg [2], and 0.25 mg [1], inhalation 0.5 mg [1], solid oral dosage form 5 mg [1], and 50 mg [1].
Thiopental	Injection 0.5 g [6], and 1 g [5].
Tinidazole	Solid oral dosage form 500 mg [4], 50 mg [1], and 250 mg [1].
Tobramycin	Eye drops 3 mg/ml [1].
Triamcinolone acetonide	No country listing acetonide for systemic use.
Tropisetron	Injection 5 mg [1], and solid oral dosage form 5 mg [1].
Vitamin d: ergocalciferol	Oral liquid 250 ug/ml (10,000 IU) [2], solid oral dosage form 1.25 mg [2], oral liquid 25 ug/5 ml [1], 5,000 IU/ml [1], and 600,000 IU/1.5 ml [1], solid oral dosage form 1,000 IU [1], 20,000 IU [1], 40,000 IU [1], and 50,000 IU [1].

## 4 Discussion

This study highlights the inclusion of pediatric formulations and medicines in national essential medicines lists (EMLs) across 158 countries, revealing significant variation in how pediatric needs are addressed. Our cross-sectional study found that most countries do not mention pediatric formulations in their EML. This problem is particularly acute in high-income countries, such as those belonging to the European region. Notably, countries in the African region demonstrated the highest inclusion of pediatric formulations, particularly lower-middle-income and low-income nations. This may suggest that countries with fewer resources may prioritize children's health through strategic listing of pediatric formulations, likely in response to public health needs and health system constraints. The disparity in the number of pediatric medicines listed across countries is also noteworthy. Some countries, like Kenya and Tunisia, displayed a stark contrast in the number of medicines listed for adults vs. children, with relatively fewer pediatric options despite having an extensive adult medicine list. Conversely, countries such as Benin and Tuvalu presented a more balanced approach, listing a similar number of medicines for both adults and children. These findings highlight the inconsistencies in national health priorities and may point to logistical, financial, or policy barriers that affect the inclusion of pediatric medicines in some regions. Furthermore, the diversity of pediatric formulations listed, particularly for medicines like paracetamol, demonstrates the importance of offering multiple formulation options to ensure accessibility and effective treatment for children. The substantial variation in formulation types for common medications, such as paracetamol, underscores the need for flexibility in providing medicines that meet the varying needs of pediatric populations across different settings.

For the 22 countries that specify pediatric formulations, most include treatments for infections, antiretrovirals and cancer medicines. There are some gaps in listing antitrypanosomal, antileishmanial, and antihepatitis therapies. There are also differences, overall and in the six therapeutic areas, between the medicines recommended by the WHO and some countries' lists, with countries including a lower number of medicines compared to the WHO, overall and compared to each therapeutic area (except for HIV and hepatitis, where some countries included more medicines than those listed in the WHO EMLc).

Although the costs of pediatric formulations may be one reason for not listing them (1), health spending is not related to the number of pediatric formulations listed and many countries with low health spending listed a relatively large number of pediatric formulations. Guidance about how and when pediatric formulations should be used is variable, and essential medicines lists are intended to be used in conjunction with other documents such as standard treatment guidelines. More research is needed to explore if national guidelines address better the selection of medicines and formulations for children. Examples of medicine formulations listed in national lists that are included in the WHO EMLc as therapeutic alternatives without specific formulation details might trigger inclusion of this additional information as part of the updates of the WHO model essential medicine list for children.

Overall, these findings emphasize the importance of tailoring national EMLs to the unique health needs of children, particularly in resource-limited settings. While significant progress has been made in some regions, further efforts are needed to standardize the inclusion of pediatric medicines and formulations in national EMLs to ensure equitable access to essential treatments for children globally.

### 4.1 Strengths and limitations

This is the largest study of pediatric formulations in EML assessing 158 countries. We use the most recent available information about essential medicines lists. However, some countries have not updated their lists in the past decade. Some of the differences between national lists and WHO EMLc could be due to these delays: 40% of the 22 countries prioritising medicines for children do not have lists published within the past five years from latest WHO EMLc 2023. We may have missed some list documents that were not available online although we used multiple methods to identify national lists. Pediatric formulations of medicines might actually be available even where they are not included in national EMLs. This cross-sectional study does not provide information about how listing has changed over time; there may be improvements not captured by our study.

## 5 Conclusions

Affirmations of the importance of child health are not reflected in EMLs. More attention to pediatric formulations of essential medicines is needed as most countries do not mention pediatric formulations in their EMLs. The presence of dedicated pediatric EMLs can help ensure that children's specific health needs are adequately addressed in national health policies. Pediatric populations often require different formulations, dosages, and types of medicines compared to adults due to their unique physiological characteristics, such as body size, metabolism, and developmental stage. Therefore, having a pediatric EML or specifying pediatric formulations in a country's general EML is crucial for ensuring access to the right medicines for children, especially in resource-limited settings. Timely updating of essential medicines for children is also vital. These findings are consistent with those of other studies, including one that highlighted the need to update India's EML for children. Notably, we found that India's pediatric EML is 11 years old, whereas the adult list was last updated in 2022 (24).

Countries can use the WHO's model list to help ensure appropriate essential medicines for children are procured and supplied. This study can also provide guidance for monitoring the progress of essential medicines' availability for the realisation of the Sustainable Development Goals and the WHO General Programme of Work framework.

## Data Availability

The raw data supporting the conclusions of this article will be made available by the authors, without undue reservation.
