# Supplementary material for: Pediatric formulations in national essential medicines lists: a cross-sectional study
Source: Front Pediatr. 2025 May 2;13:1566841. doi: 10.3389/fped.2025.1566841 (PMC12081460; doi:10.3389/fped.2025.1566841)
Supplement: Supplementary file 1 [file Datasheet1.pdf]

**Supplementary data**

***Six therapeutic areas***

*Pediatric medicines in six therapeutic areas*

We examined these 22 lists using the ATC codes to select pediatric medicines in the six therapeutic areas of interest: anti-infective medicines (antibiotics), cancer, neglected diseases (including leishmaniasis, helminthiasis and trypanosomiasis), HIV, tuberculosis, and viral hepatitis. The most frequently listed medicines included ACCESS group antibiotics (listed 293 times), cancer medicines (listed 253 times), and HIV treatments (listed 196 times). Compared to the WHO EMLc which listed a total of 142 medicines across the six therapeutic areas, Chad listed a total of 105 medicines, followed closely by Benin with 102. Tunisia is the country listing the lowest number of total medicines in the six areas, with only five.

Benin included the highest number of ACCESS group antibiotics, with 22, compared to the 19 listed in the WHO EMLc. Mexico listed 20 antibiotics from the WATCH group, while the WHO EMLc included 11. The Democratic Republic of the Congo mentioned 5 RESERVE group antibiotics, whereas the WHO EMLc included six.

Mexico also included the highest number of cancer medicines, with 31, compared to 44 in the WHO EMLc.

In the area of neglected diseases, the Democratic Republic of the Congo listed ten anthelmintics, matching the WHO EMLc's count. Nigeria listed four antitrypanosomal medicines, while the WHO EMLc included 8. Benin, Burkina Faso, Chad, and Nigeria each listed three antileishmanial medicines, compared to five in the WHO EMLc.

Burkina Faso and Chad included 19 HIV treatments and 12 antituberculosis medicines in their lists, while the United Republic of Tanzania also listed 12 tuberculosis treatments but only nine for HIV. In comparison, the WHO EMLc listed ten HIV treatments and 22 antituberculosis medicines. Additionally, Chad's essential medicine list included eight medicines for treating viral hepatitis, while the WHO included six.

*Pediatric formulations in six therapeutic areas*

When analysing the relationship of pediatric formulations listed by each country and the WHO EMLc (Figures 3, 4, 5, 6, 7, and 8), we found that antibiotics, especially those belonging to the ACCESS group, were the formulations with higher agreement between countries listing and the WHO EMLc (green color cells, Figure 3). Antibiotics from the WATCH group were the ones with more discrepancies between the formulations listed by the different countries and the WHO model list (yellow color cells, Figure 3). Cancer was the therapeutic area with more formulations recommended by the WHO but not included in the countries' lists (red color cells, Figure 4). On the contrary, medicines to treat HIV were the

ones mostly listed by countries but not by the WHO model list (white color cells, Figure 6). Sulfamethoxazole + trimethoprim was the only medicine for which all countries agreed with at least one of the WHO recommended formulations, while trimethoprim, ceftolozane + tazobactam, dasatinib, nilotinib, realgar-Indigo naturalis formulation, pegaspargase, pegfilgrastim, miltefosine, and benznidazole were not included in any country list although the WHO included them all.

*Formulations in six therapeutic areas not included in WHO EMLc*

69 medicines or combinations were cited by at least one country but were not included in the WHO EMLc. These included 22 cancer therapeutics, 22 antibiotics (comprising ten from the ACCESS group, ten from the WATCH group, and two from the RESERVE group), 17 HIV treatments, five anti-tuberculosis medicines, two hepatitis treatments, and one antihelmintic medicine (white color cells, Figures 3, 4, 5, 6, 7, and 8). Among these 69 medicines, efavirenz (which was removed from the WHO EMLc in 2021) was the most frequently mentioned, appearing in the lists of 12 countries.

## Supplementary tables

**Number of listed medicines in the six therapeutic areas of interest. Note: Countries with an asterisk are the ones with separate pediatric lists.**

| Country | Antibiotics | | | Cancer | Neglected diseases | | | HIV | Tuberculosis | Hepatitis |
|---|---|---|---|---|---|---|---|---|---|---|
| | Antibiotics ACCESS | Antibiotics WATCH | Antibiotics RESERVE | | Antihelminthics | Antileishmaniasis | Antitrypanosomal | | | |
| WHO model EMLc | 19 | 11 | 6 | 44 | 10 | 5 | 8 | 10 | 21 | 6 |
| Afghanistan | 14 | 4 | 0 | 2 | 3 | 1 | 0 | 10 | 5 | 1 |
| Bahrain | 17 | 13 | 3 | 8 | 1 | 0 | 0 | 3 | 4 | 2 |
| Bangladesh | 15 | 3 | 0 | 2 | 3 | 1 | 0 | 6 | 2 | 2 |
| Benin * | 22 | 17 | 1 | 28 | 7 | 3 | 3 | 15 | 4 | 2 |

| | | | | | | | | | | |
|---|---|---|---|---|---|---|---|---|---|---|
| Burkina Faso * | 16 | 15 | 1 | 23 | 5 | 3 | 3 | 19 | 12 | 2 |
| Chad * | 16 | 15 | 1 | 23 | 5 | 3 | 3 | 19 | 12 | 8 |
| Congo * | 13 | 8 | 4 | 15 | 6 | 0 | 2 | 12 | 9 | 1 |
| Cote D'Ivoire | 9 | 4 | 1 | 1 | 3 | 0 | 0 | 6 | 1 | 1 |
| Democratic Republic of the Congo * | 18 | 15 | 5 | 13 | 10 | 1 | 3 | 14 | 10 | 2 |
| Eswatini | 14 | 4 | 0 | 1 | 4 | 0 | 0 | 0 | 2 | 0 |
| Guinea * | 14 | 10 | 2 | 19 | 5 | 1 | 1 | 9 | 10 | 1 |
| India * | 11 | 4 | 0 | 7 | 3 | 2 | 1 | 12 | 4 | 1 |
| Kenya | 4 | 4 | 1 | 2 | 0 | 0 | 0 | 4 | 3 | 0 |
| Lesotho | 10 | 4 | 0 | 1 | 0 | 0 | 0 | 0 | 2 | 0 |
| Malawi | 12 | 6 | 0 | 4 | 4 | 1 | 2 | 7 | 6 | 0 |
| Mexico | 17 | 20 | 1 | 31 | 4 | 1 | 0 | 8 | 7 | 2 |
| Nigeria * | 14 | 11 | 0 | 27 | 7 | 3 | 4 | 12 | 10 | 3 |
| Rwanda * | 17 | 15 | 0 | 28 | 7 | 1 | 0 | 16 | 9 | 1 |
| Tunisia | 2 | 2 | 0 | 1 | 0 | 0 | 0 | 0 | 0 | 0 |
| Tuvalu * | 11 | 1 | 0 | 6 | 4 | 0 | 0 | 4 | 4 | 1 |
| United Republic of Tanzania | 15 | 12 | 2 | 10 | 3 | 1 | 0 | 9 | 12 | 4 |
| Zimbabwe | 12 | 6 | 0 | 1 | 2 | 0 | 0 | 11 | 6 | 0 |

**Medicines included in the six therapeutic areas of interest.**

| **Antibiotics** | **Cancer** |
|---|---|

| **Antibiotics AWARE** | **Antibiotics WATCH** | **Antibiotics RESERVE** | |
|---|---|---|---|
| amikacin | azithromycin | avibactam + ceftazidime | adalimumab |
| amoxicillin | cefaclor | aztreonam | antilymphocyte immunoglobulin (horse) |
| amoxicillin + clavulanic acid | cefepime | ceftolozane + tazobactam | antithymocyte immunoglobulin (rabbit) |
| ampicillin | cefixime | colistin | arsenic trioxide |
| ampicillin + sulbactam | cefotaxime | fosfomycin | asparaginase (crisantaspase recombinant) |
| benzathine penicillin G (benzathine benzylpenicillin) | cefpodoxime | linezolid | azathioprine |
| benzylpenicillin (penicillin G) | ceftazidime | meropenem + vaborbactam | basiliximab |
| cefadroxil | ceftriaxone | polymyxins | bcg vaccine |
| cefalexin | cefuroxime | | bleomycin |
| cefalotin | ciprofloxacin | | busulfan |
| cefazolin | clarithromycin | | carboplatin |
| cefradine | ertapenem | | chlorambucil |
| clindamycin | erythromycin | | ciclosporin |
| cloxacillin | fosfomycin | | cisplatin |
| dicloxacillin | imipenem + cilastatin | | cyclophosphamide |
| doxycycline | josamycin | | cytarabine |
| flucloxacillin | kanamycin | | dacarbazine |
| gentamicin | levofloxacin | | dactinomycin |
| metronidazole | lincomycin | | dasatinib |
| nitrofurantoin | meropenem | | daunorubicin |
| oxacillin | moxifloxacin | | docetaxel |
| phenoxymethylpenicillin (penicillin V) | netilmicin | | doxorubicin (adriamycin) |
| procaine benzylpenicillin | ofloxacin | | epirubicin |

| | | | |
|---|---|---|---|
| secnidazole | oxytetracycline | | etanercept |
| spectinomycin | piperacillin + tazobactam | | etoposide |
| sulfadiazine | rifaximin | | everolimus |
| sulfamethoxazole + trimethoprim | roxithromycin | | filgrastim |
| thiamphenicol | spiramycin | | fludarabine |
| tinidazole | streptomycin | | fluorouracil |
| trimethoprim | teicoplanin | | hydroxycarbamide (hydroxyurea) |
| | tobramycin | | ifosfamide |
| | vancomycin | | imatinib |
| | | | infliximab |
| | | | interferon alfa-2a |
| | | | interferon alfa-2b |
| | | | irinotecan |
| | | | leuprorelin (leuprolide) |
| | | | lomustine |
| | | | melphalan |
| | | | mercaptopurine |
| | | | methotrexate |
| | | | mifamurtide |
| | | | mitoxantrone |
| | | | mycophenolic acid (mycophenolate mofetil) |
| | | | nilotinib |
| | | | oprelvekin |
| | | | oxaliplatin |
| | | | paclitaxel |

| | | | |
|---|---|---|---|
| | | | pegaspargase |
| | | | pegfilgrastim |
| | | | procarbazine |
| | | | realgar-Indigo naturalis formulation |
| | | | rituximab |
| | | | sirolimus (rapamycin) |
| | | | temozolomide |
| | | | thiotepa |
| | | | tioguanine |
| | | | tocilizumab |
| | | | triptorelin |
| | | | vinblastine |
| | | | vincristine |
| | | | vinorelbine |

| **Neglected diseases** | | |
|---|---|---|
| **Antileishmaniasis** | **Antitrypanosomal** | **Antihelminthics** |
| meglumine antimonate | benznidazole | praziquantel |
| sodium stibogluconate | fexinidazole | oxamniquine |
| miltefosine | nifurtimox | triclabendazole |
| amphotericin b | melarsoprol | mebendazole |
| paromomycin | pentamidine | albendazole |
| | suramin sodium | flubendazole |
| | | diethylcarbamazine |
| | | pyrantel |

| | | |
|---|---|---|
| | | levamisole |
| | | ivermectin |
| | | niclosamide |

| **HIV** | **Tuberculosis** | **Hepatitis** |
|---|---|---|
| abacavir | 4-aminosalicylic acid | daclatasvir |
| abacavir + lamivudine | bedaquiline (diarylquinoline) | daclatasvir + sofosbuvir |
| abacavir + lamivudine + zidovudine | cycloserine | entecavir |
| atazanavir | delamanid | glecaprevir + pibrentasvir |
| atazanavir + ritonavir | ethambutol | lamivudine |
| darunavir | ethambutol + isoniazid | ribavirin |
| darunavir + ritonavir | ethambutol + isoniazid + rifampicin | sofosbuvir |
| dolutegravir | ethambutol + isoniazid + pyrazinamide + rifampicin | sofosbuvir + ledipasvir |
| dolutegravir + lamivudine + tenofovir | ethionamide | sofosbuvir + velpatasvir |
| efavirenz | isoniazid | tenofovir |
| efavirenz + emtricitabine + tenofovir | isoniazid + rifampicin | |
| efavirenz + lamivudine + tenofovir | isoniazid + rifapentine | |
| emtricitabine | isoniazid + pyrazinamide + rifampicin | |
| emtricitabine + tenofovir | isoniazid + pyridoxine + sulfamethoxazole + trimethoprim | |
| lamivudine | protionamide | |
| lamivudine + nevirapine + stavudine | pyrazinamide | |
| lamivudine + nevirapine + zidovudine | rifabutin | |
| lamivudine + stavudine | rifampicin | |
| lamivudine + tenofovir | rifapentine | |
| lamivudine + zidovudine | | |

| | | |
|---|---|---|
| lopinavir + ritonavir | | |
| nevirapine | | |
| raltegravir | | |
| ritonavir | | |
| stavudine | | |
| tenofovir | | |
| zidovudine (azidothymidine) | | |
